# Testing comparative phylogeographic models of marine vicariance and dispersal using a hierarchical Bayesian approach

**DOI:** 10.1186/1471-2148-8-322

**Published:** 2008-11-27

**Authors:** Michael J Hickerson, Christopher P Meyer

**Affiliations:** 1 Biology Department, Queens College, City University of New York, 65-30 Kissena Blvd, Flushing, NY 11367-1597, USA; 2 Smithsonian Institution, PO Box 37012, MRC 163, Washington, DC 20013-7012, USA

## Abstract

**Background:**

Marine allopatric speciation is an enigma because pelagic larval dispersal can potentially connect disjunct populations thereby preventing reproductive and morphological divergence. Here we present a new hierarchical approximate Bayesian computation model (HABC) that tests two hypotheses of marine allopatric speciation: 1.) "soft vicariance", where a speciation involves fragmentation of a large widespread ancestral species range that was previously connected by long distance gene flow; and 2.) peripatric colonization, where speciations in peripheral archipelagos emerge from sweepstakes colonizations from central source regions. The HABC approach analyzes all the phylogeographic datasets at once in order to make across taxon-pair inferences about biogeographic processes while explicitly allowing for uncertainty in the demographic differences within each taxon-pair. Our method uses comparative phylogeographic data that consists of single locus mtDNA sequences from multiple co-distributed taxa containing pairs of central and peripheral populations. We use the method on two comparative phylogeographic data sets consisting of cowrie gastropod endemics co-distributed in the Hawaiian (11 taxon-pairs) and Marquesan archipelagos (7 taxon-pairs).

**Results:**

Given the Marquesan data, we find strong evidence of simultaneous colonization across all seven cowrie gastropod endemics co-distributed in the Marquesas. In contrast, the lower sample sizes in the Hawaiian data lead to greater uncertainty associated with the Hawaiian estimates. Although, the hyper-parameter estimates point to soft vicariance in a subset of the 11 Hawaiian taxon-pairs, the hyper-prior and hyper-posterior are too similar to make a definitive conclusion. Both results are not inconsistent with what is known about the geologic history of the archipelagos. Simulations verify that our method can successfully distinguish these two histories across a wide range of conditions given sufficient sampling.

**Conclusion:**

Although soft vicariance and colonization are likely to produce similar genetic patterns when a single taxon-pair is used, our hierarchical Bayesian model can potentially detect if either history is a dominant process across co-distributed taxon-pairs. As comparative phylogeographic datasets grow to include > 100 co-distributed taxon-pairs, the HABC approach will be well suited to dissect temporal patterns in community assembly and evolution, thereby providing a bridge linking comparative phylogeography with community ecology.

## Background

Allopatric speciation is an enigma in many marine organisms because larval dispersal can potentially connect disjoint populations and thereby prevent the reproductive and morphological divergence that arises from prolonged isolation [1-7]. This is especially enigmatic in the Indo-Pacific region where many species range freely across this expanse without evidence for barriers to genetic exchange. This marine region harbors the planet's highest species diversity and endemism of marine fauna, and with the absence of explicit barriers, some have pushed controversial models of sympatric speciation to explain this elevated diversity [8,9]. Even the proposed competing models of geographic speciation in the Indo-Pacific remain contentious and generally involve different facets of the classic dispersal or vicariance models for speciation. Under the one model (the "soft vicariance" model), speciations in peripheral archipelagoes result from a large widespread patchy ancestral species range connected by long distance gene flow that is eventually interrupted by oceanographic changes in temperature, sea level and/or currents [10-12] which leads to peripheral isolation and endemism. Under a second model (the "colonization" model), speciations in peripheral (i.e peripatric) archipelagoes emerge from sweepstakes centrifugal colonizations from high-diversity central areas followed by prolonged periods of isolation with potential inward range shifts towards the central region [8,9,13,14]. As in the case of terrestrial systems, using genetic data to distinguish these two scenarios is difficult because their expected genetic signatures are often similar, a situation that is exacerbated if demographic changes such as expansions and bottlenecks occur after an isolating event [10,15-17].

Likewise, discerning the modes of isolation and speciation using phylogenetic and phylogeographic data is often fraught with uncertainty because species can potentially shift their ranges [18] or loose the population genetic patterns associated with colonization >> 2N generations subsequent to isolation. Traditionally, vicariance and dispersal histories have been tested using phylogenetic approaches that use area cladograms [19,20], consensus methods [21], or parsimony [22] in combination with some method of ancestral character state reconstruction. Although many of these classic methods were biased to find vicariance, recent methods incorporate more complex biogeography histories [23,24] such as maximum likelihood [25,26] and Bayesian methods that use both distributional and phylogenetic data [27]. Likewise, empirical studies have increasingly found dispersal/colonization to be a more common force behind allopatric speciation [15,16,28-31]. Regardless, such analyses are often circular or ambiguous [32], and ancestral character reconstruction methods are always going to be hindered when elevated homoplasy in biogeographic patterns obscures the inferences in the older parts of a phylogeny [18,31,33].

Here we present an entirely different approach to testing for vicariance and dispersal histories. Instead of using phylogenetic comparative methods, we use coalescent population genetics to estimate ancestral demographic patterns across co-distributed taxa within a community. While this is in the spirit of previous suggested approaches that blend systematics and population genetics [34], the hierarchical Bayesian approach presented here tests vicariance and dispersal across taxon-pairs instead of doing so one at a time. Specifically we extend a hierarchical approximate Bayesian model (HABC) [35,36] in order to quantify the strength of these two alternative models of allopatric isolation across marine endemic taxa that are co-distributed in peripheral archipelagoes.

Using HABC allows sidestepping the requirement of an explicit likelihood function. Instead, it uses a probabilistic simulation model to generate data sets to compare with the empirical data. By using summary statistics, one can easily compare the simulated and empirical data in order to estimate parameters of the simulation model via an approximate sample of the posterior distribution. In HABC we use hyper-parameters that describe processes across co-distributed taxon-pairs as well as sub-parameters that describe the demographic history of each taxon-pair.

First we describe the population genetic models whose hyper-parameters we want to estimate, and then we describe HABC. After detailing the HABC model, the summary statistics and the HABC implementation, we test these two biogeographic hypotheses given two comparative phylogeographic datasets: mtDNA CO1 data collected from multiple cowrie gastropod species that are endemic to the Hawaiian and Marquesan archipelagos. Specifically we use HABC to test whether marine vicariance ("soft vicariance") (*H*_*1*_) or colonization (*H*_*2*_) is the dominant isolating mechanism in either of these two marine communities. After using HABC to choose the best model of community isolation, we then use HABC to estimate temporal congruence in soft vicariance and/or colonization. We specifically use mtDNA sequence data collected from each co-distributed peripheral endemic taxon as well as each of the respective sister species which are usually more geographically widespread (Additional file 1).

Although this method specifically addresses questions relevant to the species diversity and patterns of endemism in the Indo-Pacific, it will be broadly applicable to many comparative phylogeographic datasets and biogeographic settings.

## Methods description

### Soft vicariance and colonization

**Figure 1:**
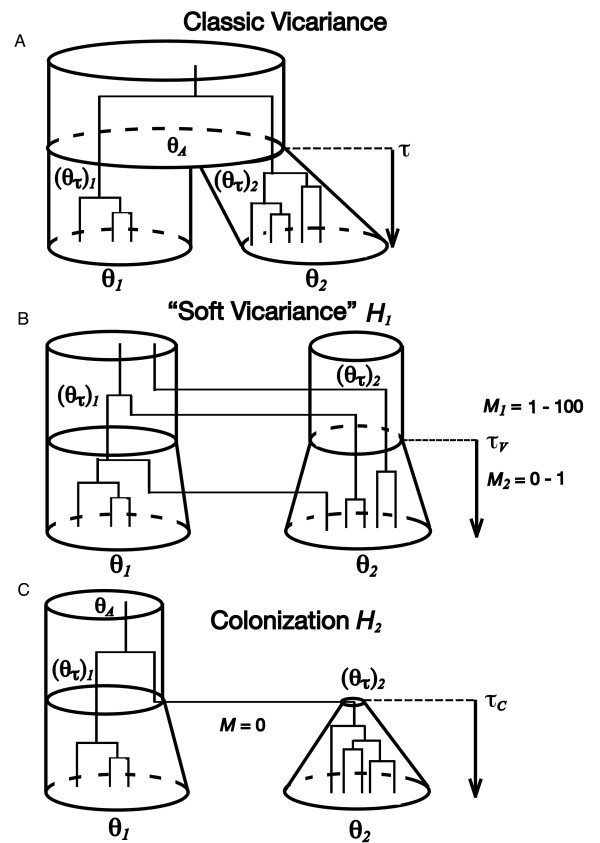
**Three models of allopatric isolation**. (A) Classic vicariance where a large ancestral population is broken up into two isolated sister populations. (B) Marine vicariance or "soft vicariance" where two ancestral populations with effective sizes (*θ*_*τ*_)_1 _and (*θ*_*τ*_)_2 _are connected by high to moderate gene flow (*M*_*1 *_= 1.0 to 100.0 migrants per generation) until *τ*_*V*_, when *M*_*1 *_decreases to 0.0 – 1.0 migrants per generation (*M*_*2*_). (C) Isolation by colonization, where one of the sister populations is founded by a very small number of individuals (*θ*_*τ*_)_2 _that come from a larger source population (*θ*_*τ*_)_1 _at the time of colonization, *τ*_*C*_.

Rather than classical terrestrial vicariance where a large ancestral population is broken up into two isolated sister populations (Figure 1A), our "soft vicariance" scenario (*H*_1_; Figure 1B) has two ancestral populations with effective sizes (*θ*_*τ*_)_1 _and (*θ*_*τ*_)_2 _that are connected by high to moderate gene flow (*M*_*1 *_= 1.0 to 100.0 migrants per generation) until *τ*_*V*_, when *M*_*1 *_decreases to 0.0 – 1.0 migrants per generation (*M*_*2*_). If this second period is prolonged, then effective isolation and divergence can occur [37]. At *τ*_*V*_, the sizes of the two sister populations ((*θ*_*τ*_)_1 _and (*θ*_*τ*_)_2_) remain the same size or begin to grow exponentially until they reach their present sizes (*θ*_*1 *_and *θ*_*2*_) at *τ *= 0 depending on the draw from the prior (Figure 1B). As in [16,17], time of vicariance, population sizes and migration rates are all free to vary across taxon-pairs according to their prior distributions.

Under the colonization scenario (*H*_*2*_; Figure 1C), one of the sister populations is founded by a very small number of individuals (*θ*_*τ*_)_2 _that come from a larger source population (*θ*_*τ*_)_1 _at the time of colonization, *τ*_*C *_with subsequent isolation. The small colonizing population (*θ*_*τ*_)_2_, then grows exponentially until it reaches its present effective size of θ_2 _at *τ *= 0. The primary parametric expectation that distinguishes marine vicariance (*H*_*1*_) from colonization (*H*_*2*_) is the relatively small effective population size of the colonized population ((*θ*_*τ*_)_2_) at the putative time of colonization (*τ*_*C*_). Secondarily, the possibility of gene flow subsequent to the vicariance event *τ*_*V *_further distinguishes *H*_*1 *_and *H*_*2*_, although this assumption can potentially be relaxed. With regards to different patterns in the molecular genetic data under *H*_*1 *_and *H*_*2*_, under colonization (*H*_*2*_) samples from peripheral populations will likely accumulate a surplus of rare alleles due to having a current effective population size that greatly expanded from a small size after the colonization time *τ*_*C*_. In addition, there is likely to be generally more genetic diversity under soft vicariance (*H*_*1*_) due to there being two ancestral populations rather than just one.

To statistically quantify the relative support of these two hypotheses (*H*_*1 *_and *H*_*2*_) across *Y *co-distributed peripheral endemics and their sister taxa given DNA sequence data, we extend and modify the hierarchical approximate Bayesian computation (HABC) framework of [35,36]. We also use this framework to estimate the temporal congruence of both vicariance and colonization across the *Y *phylogeographic data sets.

Although one could independently estimate (*θ*_*τ*_)_2 _in each of the *Y *data sets and use all of the independent posterior densities of (*θ*_*τ*_)_2 _to measure the support of *H*_*1 *_and *H*_*2 *_across the *Y *pairs, implementation of a hierarchical model accomplishes this from a single analysis and uses more information from the data via "borrowing strength" [38-40].

### Hierarchical approximate Bayesian computation

Our implementation of HABC is based on the framework presented in [35,36,41], and we review the important features here. In HABC, sub-parameters (*Φ*; within taxon-pair parameters) are conditional on "hyper-parameters" (*φ*) that quantify the variability of *Φ *among the *Y *taxon-pairs. Instead of explicitly calculating the likelihood expression P(Data|*φ*, *Φ*) to get a posterior distribution, we sample from the posterior distribution P((*φ*, *Φ*)|Data) by simulating the data *K *times under a coalescent model using candidate parameters randomly drawn from the joint hyper-prior and sub-prior distribution P(*φ*, *Φ*). A summary statistic vector **D**_*i *_for each simulated dataset is then compared to the observed summary statistic vector D* in order to generate random observations from the joint posterior distribution *f*(*φ*_*i*_, *Φ*_*i*_|**D**_*i*_) by way of a rejection/acceptance algorithm followed by a weighted local linear regression step [42].

The rejection/acceptance algorithm involves calculating a summary statistic vector from the observed data and each of the *K *simulated data sets. Each simulated data set is generated using parameters that are randomly drawn from the joint prior. Following [35,42], *K *Euclidian distances between the normalized observed summary statistic vector D* and each of the *K *normalized summary statistic vectors are then calculated (||**D**_*i *_- D*|| = *d*). An arbitrary proportion (tolerance) of the *K *simulations with the lowest *d *values are then used to obtain an approximate sample from the joint posterior after weighting and transforming the accepted parameter values using local linear regression [35,42]. After the local linear regression step, accepted and transformed parameter values that fall outside their respective prior bounds are subsequently transformed to have the values of their respective prior boundaries. For example, if an accepted parameter value with a uniform prior of [0.0,1.0] is transformed by local linear regression to a negative number, it is subsequently transformed to 0.0.

### Hierarchical Model of Community Colonization and Vicariance

Model hyper-parameters and sub-parameters are listed and described in Additional file 2. Three hyper-parameters are drawn from their respective hyper-prior distributions (Additional file 2A), and these include: 1.) *Z*, the number of descendent populations per *Y *taxon-pairs that arise by colonization at times $T_{C}=\{\tau_{C}^{1},...,\tau_{C}^{Z}\}$; 2.) the number of *different *vicariance times ${\bar{\Psi }}_{V}=\{t_{V}^{1},...,t_{V}^{\Psi_{V}}\}$ across (*Y-Z) actual *vicariance times $T_{V}=\{\tau_{V}^{1},...,\tau_{V}^{\left(Y-Z\right)}\}$ and 3.) Ψ_*C*_, the number of *different *colonization times ${\bar{\Psi }}_{C}=\{t_{C}^{1},...,t_{C}^{\Psi_{C}}\}$ across *Z actual *colonization times $T_{C}=\{\tau_{C}^{1},...,\tau_{C}^{Z}\}$.

The *Z *colonized populations (*H*_*2*_) and remaining (*Y*-*Z*) population-pairs that arise via vicariance (*H*_*1*_) then draw their population mutation sub-parameters ($\vec{\theta }$ = {*θ*^1^,..., *θ*^*Y*^}, ${\vec{\theta }}_{1}=\{\theta_{1}^{1},...,\theta_{1}^{Y}\}$, and ${\vec{\theta }}_{2}=\{\theta_{2}^{1},...,\theta_{2}^{Y}\}$) from their respective sub-priors (Additional file 2C). Each taxon-pair's population mutation parameter *θ*^*i *^is equal to the sum of $\theta_{1}^{i}$ and $\theta_{2}^{i}$, the population mutation parameters of the descendent taxon-pairs at *τ *= 0 (present time). In this case *θ *= 2*Nμ *(2*N *is the sum of the two haploid effective female population sizes of each pair of descendent populations and *μ *is the per gene per generation mutation rate).

Subsequently, each of the *Y *taxon-pairs draw their remaining sub-parameters from two different sets of sub-priors (Additional file 2D) that differentially characterize the two different histories (*H*_*1 *_and *H*_*2*_). Importantly, the uniform sub-prior for (*θ*_*τ*_)_2 _is [0.0, 0.05] for each of the *Z *species-pairs that arose via colonization (*H*_*2*_), but (*θ*_*τ*_)_2 _is drawn from the uniform sub-prior [0.0, 1.0] for each of the other (*Y *- *Z*) taxon-pairs that arose through vicariance (*H*_*1*_). Additionally, two sets of migration sub-parameters (${\vec{M}}_{1}=\{M_{1}^{1},...,M_{1}^{\left(Y-Z\right)}\}$ and ${\vec{M}}_{2}=\{M_{2}^{1},...,M_{2}^{\left(Y-Z\right)}\}$) are drawn from their respective sub-priors for the (*Y *- *Z*) taxon-pairs that arose through vicariance (*H*_*1*_), whereas there is no migration under the colonization model (Figure 1C; Additional file 2D). In both vicariance and colonization models, the relative effective size of the ancestral central populations (${\left({\vec{\theta }}_{\tau }\right)}_{1}=\{{\left(\theta_{\tau }\right)}_{1}^{1},...,{\left(\theta_{\tau }\right)}_{1}^{\left(Y-Z\right)}\}$ and ${\left({\vec{\theta }}_{\tau }\right)}_{1}=\{{\left(\theta_{\tau }\right)}_{1}^{1},...,{\left(\theta_{\tau }\right)}_{1}^{Z}\}$) are also drawn from their respective sub-priors [0.5, 1.0]. If (*Y *- *Z*) ≥ 1, the Ψ_*V *_*different *vicariance times (${\vec{\Psi }}_{V}=\{t_{V}^{1},...,t_{V}^{\Psi_{V}}\}$) are drawn from the uniform prior [0.0, 5.0]. Likewise, if *Z *≥ 1, the Ψ_*C *_*different *colonization times (${\bar{\Psi }}_{C}=\{t_{C}^{1},...,t_{C}^{\Psi_{C}}\}$) are drawn from the uniform prior [0.0, 5.0]. After the ${\vec{\Psi }}_{V}$*different *viciarance times are drawn, they are randomly assigned to the (*Y - Z*) taxon-pairs that arose through vicariance, such that the (*Y - Z*) *actual *vicariance times are $T_{V}=\{\tau_{V}^{1},...,\tau_{V}^{\left(Y-Z\right)}\}$. Specifically, the Ψ_*V *_*different *vicariance times ($t_{V}^{1},...,t_{V}^{\Psi_{V}}$) are sequentially assigned to the first Ψ_*V *_*actual *times $\tau_{V}^{1},...,\tau_{V}^{\Psi_{V}}$. The remaining actual times ($\tau_{V}^{\left(\Psi_{V}+1\right)},...,\tau_{V}^{\left(Y-Z\right)}$) are assigned by randomly drawing with replacement from the ${\vec{\Psi }}_{V}$ matrix of *different *times {$t_{V}^{1},...,t_{V}^{\Psi_{V}}$}. Likewise, the *actual *colonization times ($T_{C}=\{\tau_{C}^{1},...,\tau_{C}^{Z}\}$) are drawn using the same method (Additional file 2D). Both sets of *actual *vicariance and *actual *colonization times (*T*_*V *_and *T*_*C*_) are in units of *θ*^*i*^/μ generations, where *θ*^*i *^is each taxon-pair's population mutation parameter and μ is the per gene per generation mutation rate.

In addition to hyper-parameter estimation, we also use the HABC algorithm to sample from the posterior distributions of sub-parameter summaries (Additional file 2E) in order to quantify the support for *H*_*1 *_and *H*_*2 *_and secondarily estimate levels of temporal congruence in colonization and/or soft vicariance. Namely, we obtain estimates of the arithmetic means of three sub-parameters (*E*((*θ*_*τ*_)2), E(*τ*_*C*_), E(*τ*_*V*_)), as well as the dispersion indexes of *τ*_*C *_and *τ*_*V *_(Ω_*C *_and Ω_*V *_respectively). The sub-parameter summary E((*θ*_*τ*_)_2_) is expected to be < 0.05 if H_2 _is dominate across the Y taxon-pairs (Z = Y). The dispersion indexes Ω_*C *_and Ω_*V *_of the *Z *colonization times ($\tau_{C}^{1},...,\tau_{V}^{Z}$) and the (*Y *- *Z*) vicariance times ($\tau_{V}^{1},...,\tau_{V}^{\left(Y-Z\right)}$) measure the ratio of the variance to the mean of these two sets of times and are therefore expected to be ≈ 0.0 when if there is temporal congruence in colonization or soft vicariance.

### Two Stage Model Implementation

We implement the hierarchical analysis in two stages. In stage 1, an unconstrained general model is used to quantify the support for *H*_*1 *_and *H*_*2 *_across the *Y *taxon pairs. This first stage is accomplished by simulating *K *random draws from a general hyper-prior and using the HABC algorithm to sample from the posteriors of? *Z *and *E*((*θ*_*τ*_)_2_). Under our hierarchical model, *H*_*1 *_and *H*_*2 *_are equally probable because *Z *is a hyper-parameter drawn from the discrete uniform hyper-prior distribution [0, *Y*].

In the stage 2 analysis, *K *random draws are taken from a constrained hyper-prior where *Z *is fixed to be the mode of its posterior distribution obtained in stage 1. There are equal numbers of hyper-prior draws (*K*) obtained in both stages. The stage 2 analysis allows obtaining posterior samples of hyper-parameters and sub-parameter summaries (Additional file 2B &2E) that quantify and summarize the levels of temporal concordance in colonization ($T_{C}=\{\tau_{C}^{1},...,\tau_{C}^{Z}\}$) and/or vicariance ($T_{V}=\{\tau_{V}^{1},...,\tau_{V}^{\left(Y-Z\right)}\}$). These include: 1.) Ψ_*C*_, the number of *different *colonization times per *Z *colonization events; 2.) Ψ_*V*_, the number of *different *vicariance times per (*Y - Z*) vicariance events; 3.) Ω_*C*_, the dispersion index of *τ*_*C *_(the ratio of the variance to the mean in the *Z actual *colonization times, *T*_*C*_); and 4.) Ω_*V*_, the dispersion index of *τ*_*V *_(the ratio of the variance to the mean in (*Y - Z*) *actual *vicariance times, *T*_*V*_).

### Summary Statistic Vector for HABC Acceptance/Rejection Algorithm

In order to implement the HABC procedure, we use two modified versions of the summary statistic vector **D **used in [35]. For the Marquesas summary statistic vector (**D**_*Marquesas*_), we calculate eight summary statistics collected from each taxon-pair (56 total). This includes *π*_*b *_(average pairwise differences between each central and peripheral Marquasan taxon-pair), *π *(average pairwise differences among all individuals within each taxon-pair), *π*_*w *_(average pairwise differences within descendent populations of each taxon-pair), *θ*_*W *_(Watterson's estimator of *θ *of each taxon-pair), and Var(*π *- *θ*_*W*_). For *π*, *θ*_*W *_and Var(*π *- *θ*_*W*_), each of the *Y *taxon-pairs are treated as a single population sample (${\vec{n}}_{1}+{\vec{n}}_{2}$). The Marquesas summary statistic vector, **D**_*Marquesas *_also includes calculating *π*, *θ*_*W*_, and Var(*π *- *θ*_*W*_) in each of the *Y *peripheral Marquasan samples (${\vec{n}}_{2}$) that are putatively colonized and we denote these as *π*_2_, (*θ*_*W*_)_2_, and Var(*π *- *θ*_*W*_)_2_. Under this scheme, the vector **D**_*Marquesas *_is

$$D_{Marquesas}=\left\{\begin{array}{cccccccc} \pi^{1} & \pi_{w}^{1} & \theta_{W}^{1} & {\text{Var(}\pi -\theta_{W}\text{)}}^{1} & \pi_{2}^{1} & {\left(\theta_{W}\right)}_{2}^{1} & {\text{Var(}\pi -\theta_{W}\text{)}}_{2}^{1} & \pi_{b}^{1}\\ . & . & . & . & . & . & . & .\\ . & . & . & . & . & . & . & .\\ . & . & . & . & . & . & . & .\\ \pi^{Y} & \pi_{w}^{Y} & \theta_{W}^{Y} & {\text{Var(}\pi -\theta_{W}\text{)}}^{Y} & \pi_{2}^{Y} & {\left(\theta_{W}\right)}_{2}^{Y} & {\text{Var(}\pi -\theta_{W}\text{)}}_{2}^{Y} & \pi_{b}^{Y} \end{array}\right\},$$

where each of the *Y *rows correspond to the *Y *taxon-pairs (*Y *= 7) and the eight columns correspond to the eight summary statistic classes. After these 8 × *Y *summary statistics are calculated, we must choose a way to consistently order the *Y *rows within **D**_*Marquesas*_. Instead of consistently ordering the rows by each taxon-pair's sample size, we increase the efficiency of our HABC estimator by ordering the rows based on the taxon-pair's Tajima's D [43] calculated from the taxon-pair's peripheral population sample (${\vec{n}}_{2}$). For example, row 1 would contain the eight summary statistics collected from the taxon-pair with the lowest Tajima's D, and the *Y*^*th *^row would contain the eight summary statistics collected from the taxon-pair with the highest Tajima's D.

The motive for this ordering procedure is to extract more information from the data with respect to the estimated hyper-parameters than would be accomplished by ordering consistently by sample size [35]. For an efficient HABC estimator, there should be a strong correlation between pair-wise differences in hyper-parameter values (i.e. E(*θ*_*τ*_)_2 _or *Z*) and Euclidian distances between corresponding pairs of summary statistic vectors from corresponding pairs of simulated data sets. If ordering by sample size rather than ranked values of an informative summary statistic, pair-wise values of *Z or *E(*θ*_*τ*_)_2 _are not predicted to correlate with Euclidian distances of **D **calculated from corresponding pairwise simulated data sets. This is because sample size has no bearing on how each of the *Y *taxon-pairs are assigned to histories *H*_*1 *_and *H*_*2 *_when drawing values of *Z *from the hyper-prior. On the other hand, ordering by an informative summary statistic will minimize Euclidian distances among data sets with equal or similar values of *Z *regardless of which of the *Y *taxon pairs were assigned histories *H*_*1 *_and *H*_*2*_. The consequent improved accuracy in HABC estimation that results from this ordering procedure is based on the *exchangeability *of the *Y *rows within **D**_*Marquesa *_(**D**_1_,...,**D**_*Y*_). If *Φ*_*i *_and **D**_*i *_are invariant to the permutations of the indexes (1 ,..., *Y*) and the *i*^*th *^taxon-pair's sample size is unrelated to the expectation of its ***Φ ***_*i *_or **D**_*i*_, there is *exchangeability *in the model [44]. We order by the peripheral Tajima's D because it is a summary statistic that is predicted to be informative with respect to demographic parameter differences between histories *H*_*1 *_and *H*_*2 *_(i.e. the ratio of each taxon-pair's (*θ*_*τ*_)_2 _and *θ*_2_).

For the analysis of the 11 Hawaiian taxon-pairs, we use a reduced summary statistic vector **D**_*Hawaii *_to avoid null values that would arise in population samples that only included one individual (Additional file 1B). The vector **D**_*Hawaii *_in this case is

$$D_{Hawaii}=\left\{\begin{array}{cccc} \pi^{1} & \theta_{W}^{1} & {\text{Var(}\pi -\theta_{W}\text{)}}^{1} & \pi_{b}^{1}\\ . & . & . & .\\ . & . & . & .\\ . & . & . & .\\ {\left(\pi \right)}^{Y} & \theta_{W}^{Y} & {\text{Var(}\pi -\theta_{W}\text{)}}^{Y} & \pi_{b}^{Y} \end{array}\right\},$$

and the rows were likewise sorted by Tajima's D calculated from the peripheral populations.

## Application on real data sets

### Two Comparative Phylogeographic Implementations

We use this HABC method on two comparative phylogeographic data sets of Pacific cowrie gastropods (Cypraeidae). The first dataset consists of seven sister taxon-pairs of cowries that each consist of a descendent endemic species or sub-species that is distributed within the peripherally located Marquesas archipelago as well each sister taxon that is more geographically widespread (Additional file 1A). The second dataset consists of eleven sister taxon-pairs of cowries co-distributed within the peripherally located Hawaiian archipelago. Like in the former data set, each pair consists of a Hawaiian endemic and a more widespread sister (Additional file 1B). Both data sets consisted of 614 base pairs of the CO1 mtDNA locus collected from 2–93 individuals per taxon-pair (Additional file 1). The HABC procedure was implemented using a modified version of the MSBAYES comparative phylogeographic software pipeline [35,36] consisting of several C and R programs that are run with a Perl "front-end" and utilizes a finite sites version of Hudson's classic coalescent simulator [45]. For both analyses, 2,000,000 random draws were sampled from the hyper-prior and 1,000 – 2,000 accepted draws were used to construct hyper-posterior samples (tolerance of 0.0005 and 0.001 respectively) using the HABC acceptance/rejection algorithm.

The prior bounds for hyper-parameters and sub-parameters are given in Additional file 2. To explore the sensitivity of using different prior assumptions, we used two different upper bounds of *θ *(*θ*_*MAX *_= 25.0 and 50.0 for the Marquesas data; *θ*_*MAX *_= 50.0 and 100.0 for the Hawaiian data). These values correspond to 2× to 4× the range of within species *θ *estimates, where the average number of pairwise differences was used as an estimator of each species specific *θ *[46]. To further explore how sensitive results are to model assumptions, we alternatively ran the stage 1 analysis with the post-colonization migration prior allowed to be [0.0, 1.0] instead of zero under the colonization model (*H*_*2*_), as well as allowing post-isolation migration (*M*_2_) to be zero under the soft vicariance model (*H*_*1*_).

We calculate Bayes factors to compare the relative hyper-posterior support of either history (soft vicariance and colonization) being dominate across all *Y *taxon pairs (e.g *Z *= *0 *or *Z *= *Y*) against all other scenarios including mixed scenarios. We accomplish this by comparing relative hyper-posterior support of these two scenarios while accounting for the relative hyper-prior support for these two scenarios [47]. To calculate this Bayes factor, we use an arbitrary partition of hyper-parameter space to delineate where *H*_*1 *_or *H*_*2 *_is dominate across all *Y *taxon-pairs. For example, to evaluate the evidence of colonization being dominate across all *Y *taxon pairs (*Z *= *Y*) against all other scenarios (*Z *<*Y*), the approximate Bayes factor *B*(*Z *= *Y*, *Z *<*Y*) is the ratio of the two approximate hyper-posteriors of these two scenarios divided by the ratio of the two hyper-priors of these two scenarios,

*B*(*Z *= *Y*, *Z *<*Y*) = (*P*(*Z *= *Y*|**D **= D*)/*P*(*Z *<*Y*|**D **= D*))/(*P*(*Z *= *Y*)/*P*(*Z *<*Y*))

Alternately, we examine these two scenarios by using an arbitrary partition of *E*((*θ*_*τ*_)_2_) such that *E*((*θ*_*τ*_)_2_) = 0.05 represents a scenario where colonization is dominant across all *Y *taxon pairs, and *E*((*θ*_*τ*_)_2_) > 0.05 represents all other scenarios. In this case, the approximate Bayes factor is

$$B(E({\left(\theta_{\tau }\right)}_{2})\leq 0.05,E({\left(\theta_{\tau }\right)}_{2})>0.05)=\frac{(P(E({\left(\theta_{\tau }\right)}_{2})\leq 0.05|D=D^{*})/P(E({\left(\theta_{\tau }\right)}_{2})>0.05|D=D^{*}))}{\left(P(E({\left(\theta_{\tau }\right)}_{2})\leq 0.05\right)/P(E({\left(\theta_{\tau }\right)}_{2})>0.05))}.$$

Evaluating the evidence of soft vicariance being dominant across all *Y *taxon-pairs (*Z *= 0) against all other scenarios (*Z *> 0) is identically accomplished by calculating the two corresponding Bayes factors *B*(*Z *= 0, *Z *> 0) and *B*(*E*((*θ*_*τ*_)_2_) > 0.05, *E*((*θ*_*τ*_)_2_) ≤ 0.05). To calculate each Bayes factor, we use the accepted hyper-parameter values from the hyper-posterior sample and the random draws from the hyper-prior.

### Marquesas Results

**Figure 2:**
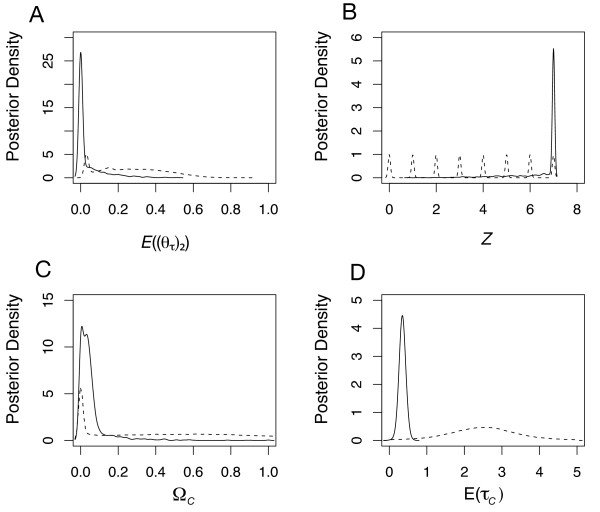
**Hyper-posterior and Hyper-prior samples given Marquesas DNA sequence data (seven taxon-pairs)**. Dashed lines depict hyper-prior distributions and solid lines depict hyper-posterior distributions. **Stage 1 hyper-parameter estimates**: (A) Average effective population size *E*((*θ*_*τ*_)_2_) of the putatively colonized endemic populations at the seven isolation times (*T*_*C *_and *T*_*V*_). (B) The number of descendent populations per seven (*Y*) taxon-pairs that arise by colonization at times $T_{C}=\{\tau_{C}^{1},...,\tau_{C}^{Z}\}$. **Stage 2 hyper-parameter estimates where hyper-prior is conditional given constant value of *Z *= 7**: (C) Ω_*C*_,dispersion index of *Z *= 7 colonization times; Ω_*C *_= Var(*τ*_*C*_)/E(*τ*_*C*_) where *τ*_*C *_is colonization time. (D) E(*τ*_*C*_), average colonization time across *Z *= 7 colonization times. For each estimate, tolerance was 0.001 (2,000 accepted draws) using the local regression algorithm.

The HABC analysis yielded a hyper-posterior that strongly supports *H*_*2*_, where colonization histories (Figures 2A &2B) are inferred across all seven Marquesan endemics. Irrespective of tolerance (0.0005 or 0.001), prior assumptions on the upper bound of θ, (*θ*_*MAX*_), the hyper-parameter mode estimates of *Z *and *E*((*θ*_*τ*_)_2_) were 7.00 and 0.00 – 0.07 respectively (Figures 2A &2B; Additional file 3A &3B). Furthermore, when using the raw untransformed accepted values to obtain our posterior sample of *Z*, a history of community colonization is also inferred (mode estimate of *Z *is 7.0) albeit the 95% credibility intervals were wider. This suggests that the dominant mode of speciation has generally been from colonization by a small number of individuals followed by at least a 20-fold demographic expansion to current population levels. Both Bayes factors evaluating the evidence for colonization across all *Y *= 7 taxon-pairs against all other histories (*Z *< 7) indicate moderate support for the former scenario (*B*(*Z *= *Y*, *Z *<*Y*) = 6.23; *B*(*E*((*θ*_*τ*_)_2_) > 0.05, *E*((*θ*_*τ*_)_2_) ≤ 0.05) = 6.79).

In stage 2, where we constrained the hyper-parameter *Z *to be 7, the hyper-posterior best supported temporal concordance in colonization across all seven Marquesas endemics. In this case, estimates of Ψ_*C *_and Ω_*C *_were 1.00 (95% quantiles: 1.00 – 2.14) and 0.00 (95% quantiles: 0.00 – 0.17) respectively (Additional file 3A; Figure 2). The mean time of colonization E(*τ*_*C*_) was 1.58 My ago if we assume a 1% divergence rate per My (Figure 2D). The Bayes factor evaluating the evidence for simultaneous colonization (Ω_*C *_< 0.05) against non-simultaneous colonization (Ω_*C *_= 0.05) yielded strong support for simultaneous colonization (*B*(Ω_*C *_< 0.05, Ω_*C *_= 0.05) = 36.73). In this case the Bayes factor is calculated from an arbitrary partition of hyper-posterior space conditional on *Z *= 7. In this case the arbitrary threshold of simultaneous colonization is Ω_*C *_< 0.05 such that the Bayes factor is

*B*(Ω_*C *_< 0.05, Ω_*C *_≥ 0.05) = (*P*(Ω_*C *_< 0.05|**D **= D*)/*P*(Ω_*C *_≥ 0.05|**D **= D*))/(*P*(Ω_*C *_< 0.05)/*P*(Ω_*C *_≥ 0.05)).

Furthermore, the hyper-posterior estimates were not sensitive to assumptions about post-isolation migration. Under the stage 1 analysis, estimates of *Z *and *E*((*θ*_*τ*_)_2_) were 6.99 and 0.02 (95% credibility intervals of 3.34 – 7.00 and 0.00 – 0.32) when the post-colonization migration prior was [0.0, 1.0] instead of 0.0 under the colonization model (*H*_*2*_).

### Hawaii Results

**Figure 3:**
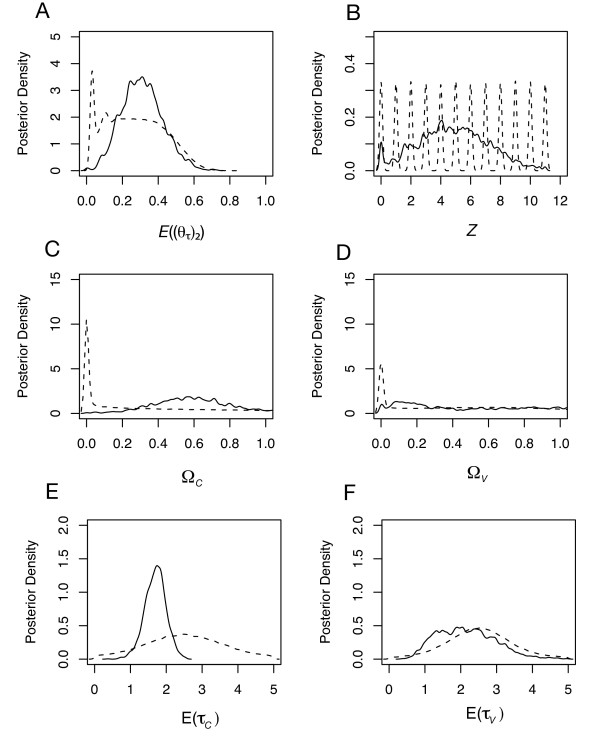
**Hyper-posterior and Hyper-prior samples given Hawaiian DNA sequence data (11 taxon-pairs)**. Dashed lines depict hyper-prior distributions and solid lines depict hyper-posterior distributions. **Stage 1 hyper-parameter estimates**: (A) Average effective population size *E*((*θ*_*τ*_)_2_) of the putatively colonized endemic populations at times the 11 isolation times (*T*_*C *_and *T*_*V*_) across the seven (*Y*) taxon-pairs. (B) The number of descendent populations per seven (*Y*) taxon-pairs that arise by colonization at times $T_{C}=\{\tau_{C}^{1},...,\tau_{C}^{Z}\}$. **Stage 2 hyper-parameter estimates where hyper-prior is conditional given constant value of *Z *= 4**: (C) Ω_*C*_,dispersion index of *Z *= 4 colonization times; Ω_*C *_= Var(*τ*_*C*_)/E(*τ*_*C*_) where *τ*_*C *_is colonization time. (D) Ω_*V*_,dispersion index of *Z *= 7 soft vicariance times; Ω_*V *_= Var(*τ*_*V*_)/E(*τ*_*V*_) where *τ*_*V *_is soft vicariance time time. (E) E(*τ*_*C*_), average colonization time across *Z *= 4 colonization times. (F) E(*τ*_*V*_), average soft vicariance time across 7 soft vicariance times. For each estimate, tolerance was 0.001 (2,000 accepted draws) using the local regression algorithm.

In contrast to the Marquesas analysis, the hyper-posteriors were much more similar to the hyper-priors (Figure 3). The less informative posteriors are consistent with the lower Hawaiian sample sizes (Additional file 1B) and consequence of using of a reduced number of summary statistics (**D**_*Hawaii*_) for the HABC acceptance/rejection algorithm. Although the hyper-posterior of *Z *given the Hawaiian data suggests a mixed history of both colonization and soft vicariance (Figure 3), larger sample sizes will be required to verify this. This weak inference is demonstrated by Bayes factors giving weak support for vicariance or colonization across all 11 Hawaiian endemics. Specifically, the calculated Bayes factors *B*(*Z *= 0, *Z *> 0), *B*(*Z *= 11, *Z *< 11), *B*(*E*((*θ*_*τ*_)_2_) < 0.05, (*E*((*θ*_*τ*_)_2_) = 0.05) < 1.0) and *B*(*E*((*θ*_*τ*_)_2_) = 0.05, *E*((*θ*_*τ*_)_2_) < 0.05) < 1.0) where all weak (< 1.0). Although the Hawaiian data yielded weak inference, the credibility intervals for *Z *ranged from 0.00 to 9.22 (Additional file 3C and 3D), suggesting that the history of isolation likely involved both soft vicariance and colonization. Likewise, estimates of Ω_*C *_and Ω_*V *_did not suggest simultaneous vicariance or colonization, and likewise yielded less informative posteriors than obtained in the Marquesas analysis (Figures 3C &3D). As was the case of the Marquesas analysis, hyper-posterior estimates were not sensitive to tolerance, prior assumptions on the upper bound of *θ*, (Additional file 3C &3D), and prior assumptions of post-isolation migration. Under the stage 1 analysis using the alternative model assumptions where post-vicariance migration (*M*_2_) was 0.0 under the soft vicariance model, estimates of *Z *and *E*((*θ*_*τ*_)_2_) were respectively 3.64 and 0.66 (95% credibility intervals 0.00 – 9.74 and 0.35 – 0.97).

## Simulation testing

### Simulated Data Sets

One of the chief advantages of HABC and ABC methods is the ease at which one can evaluate the performance, bias, and precision of the estimator via simulations. Specifically, we can simulate pseudo-observed data sets with known hyper-parameter values and compare estimates with their true values. Even though the most time-consuming task is to simulate a large enough prior and/or hyper-prior in ABC and HABC, once it is produced for the analysis of the real observed data set, it can subsequently be used to quantify bias and precision on the pseudo-observed data sets.

To this end, we simulate pseudo-observed data sets with known values of *Z *and *E*((*θ*_*τ*_)_2_) under the general model (stage 1), and Ψ_*C*_, Ψ_*V*_, E(*τ*_*C*_), E(*τ*_*V*_), Ω_*C*_, and Ω_*V *_under the constrained model (stage 2). We repeat this for both sample sizes used in the empirical implementation (**D**_*Marquesas *_and **D**_*Hawaii*_). To simulate a data set (pseudo-observed data), all hyper-parameters and sub-parameters were randomly drawn from the prior and a corresponding **D**_*Marquesas *_or **D**_*Hawaii *_was subsequently calculated. For each corresponding pseudo-observed **D**_*Marquesas *_and **D**_*Hawaii*_, a hyper-posterior sample was obtained from the HABC rejection-sampling algorithm given 2,000,000 random draws from the hyper-prior. For every pseudo-observed (simulated) data set, an estimate is obtained from the posterior mode. We report estimates using a tolerance of 0.001 corresponding to 2,000 accepted draws from the hyper-prior. For both **D**_*Marquesas *_and **D**_*Hawaii *_and stage 1 and two, 250 estimates were made from 250 simulated pseudo-observed datasets.

In addition to these evaluations, we assess the ability to distinguish *H*_*1 *_and *H*_*2 *_when the true history is a special asymmetrical case of soft vicariance (*H*_*1*_). To this end, we obtain HABC estimates of *Z *and *E*((*θ*_*τ*_)_2_) on 250 pseudo-observed datasets of size **D**_*Marquesas *_simulated under *H*_*1 *_where true values of *Z *and *E*((*θ*_*τ*_)_2_) are 0 and 0.0 – 0.05 respectively. Estimates for *Z *and *E*((*θ*_*τ*_)_2_) were obtained using a general prior (stage 1).

Another factor we explored was three other methods of post-acceptance transformation other than local linear regression (LLR) for obtaining a sample of the hyper-posterior distribution of the *Z *hyper-parameter. Because *Z *is a discrete integer (ranging from 0 to *Y*), it might be most appropriate to preserve *Z *as a discrete integer when using an ABC regression technique that implements polychotomous logit regression (PLR) [48-50]. We therefore used simulations to compare the effectiveness of LLR with: 1. PLR; 2. using the raw accepted values (RAW); and 3. a cumulative logit regression model (CLR). To compare the estimator bias and precision of these four methods, we calculated the root mean square error (RMSE) from 1000 estimates using each of these four methods on 1000 simulated pseudo-observed data sets with parameters randomly drawn from the priors. The methods for CLR and PLR are implemented in the VGAM package distributed by T. Yee under R http://www.stat.aukland.ac.nz/~yee. The LLR method is implemented from R functions made available by M. Beaumont.

### Results of Simulation Testing

**Figure 4:**
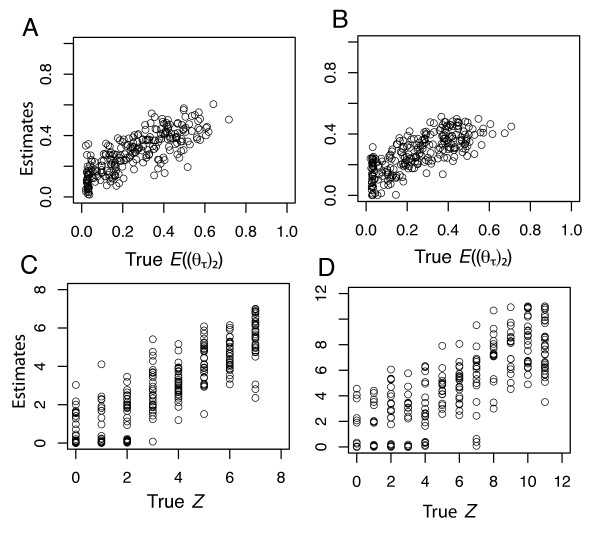
**Estimator Performance: 250 true hyper-parameter values plotted against their posterior mode estimates (stage 1 model)**. Panels (A) and (C) are given samples sizes that are identical to the Marquesas sample sizes. Panels (B) and (D) are given samples sizes that are identical to the Hawaiian sample sizes. For each estimate, tolerance was 0.001 (2,000 accepted draws) using the local linear regression algorithm.

Our simulations identified conditions under which the HABC estimator is reliable as well as conditions under which it is less reliable. At stage 1 of the HABC analysis, the estimates of *E*((*θ*_*τ*_)_2_) were consistently close to the corresponding true values of *E*((*θ*_*τ*_)_2_), although the larger sample sizes matching the Marquesas data set (**D**_*Marquesas*_) yielded more accurate estimates of *E*((*θ*_*τ*_)_2_) than using sample sizes matching the smaller Hawaii data set (**D**_*Hawaii*_; Figures 4A &4B). On the other hand, estimates of *Z *were less accurate, yet using **D**_*Marquesas *_resulted in more accurate estimates of *Z *than when using **D**_*Hawaii *_(Figures 4C &4D). Additionally, estimates of *Z *representing colonization across all *Y *taxon-pairs never resulted in false positives given **D**_*Marquesas *_(Figure 4C). Specifically, when estimates of *Z *were 6 or 7, true *Z *values were always 6 or 7. Conversely, lower true values of *Z *yielded less accurate estimates of *Z *(Figures 4C &4D). For example, when estimates of Z were 0, true values ranged from 0 – 4 given **D**_*Marquesas *_(Figure 4C). In general, the simulation analysis verified the statistical confidence in the empirical inference of colonization across all seven Marquesas endemics, yet demonstrated there to be more uncertainty in the inferred history of the 11 Hawaiian endemics.

**Figure 5:**
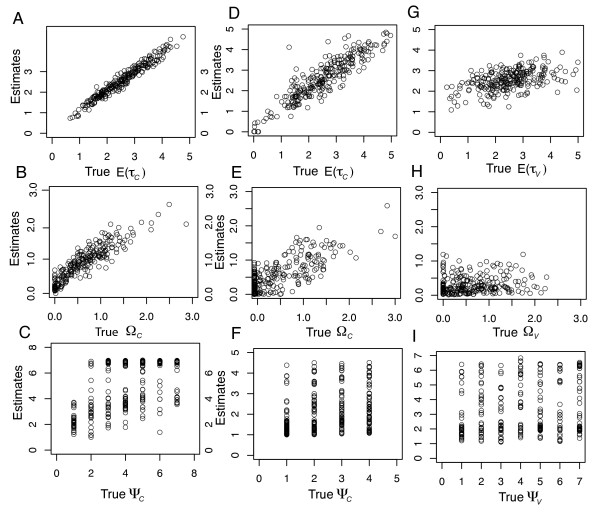
**Estimator Performance: 250 true hyper-parameter values plotted against their posterior mode estimates (stage 2 model)**. Panels (A), (B) and (C) are given samples sizes that are identical to the Marquesas sample sizes and are obtained with the hyper-prior of *Z *fixed at 7 (stage 2 model). Panels (D) through (I) are given samples sizes that are identical to the Hawaiian sample sizes and are obtained with the hyper-prior of *Z *fixed at 4 (stage 2 model). For each estimate, tolerance was 0.001 (2,000 accepted draws) using the local linear regression algorithm.

Under the constrained stage 2 model, the simulations revealed a strategy for estimating the variability in the *Z *colonization times. Specifically, the best strategy would be to use estimates of Ω_*C *_rather than Ψ_*C *_(Figure 5). However, there was less accuracy and precision in the Ω_*C *_estimates given **D**_*Hawaii *_(Figure 5E). Unlike estimates of Ω_*C*_, estimates of Ω_*V *_or E(*τ*_*V*_) were not as reliable (Figure 5H), perhaps due to the very small amount of migration (0 to 1.0 migrants per generation) after each "soft vicariance" event (*τ*_*V*_) under *H*_*1*_.

**Figure 6:**
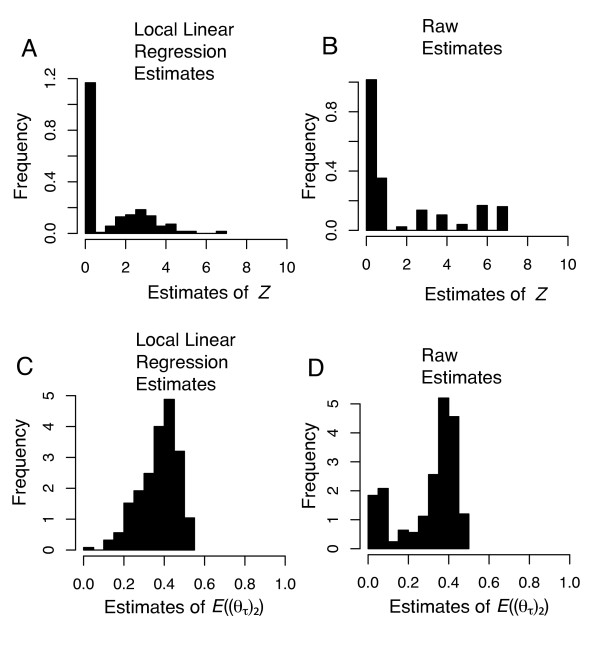
**Estimator performance under constrained history of asymmetrical soft vicariance (True *Z *= 0; True *E*((*θ*_*τ*_)_2_) < 0.05; stage 1 model)**. Each panel depicts a frequency histogram of 250 mode estimates of *Z *(A and B) and *E*((*θ*_*τ*_)_2_) (C and D) under a constrained history of asymmetrical soft vicariance where *Z *= 0 and *E*((*θ*_*τ*_)_2_) < 0.05. For all mode estimates, tolerance was 0.001 (2,000 accepted draws). Mode estimates for panels (A) and (C) are obtained using the local linear regression algorithm, whereas the mode estimates for panels (B) and (D) are obtained using the raw 2,000 accepted values.

The simulations also revealed that we have the ability to distinguish *H*_*1 *_and *H*_*2 *_when the true history is a special asymmetrical case of vicariance (*H*_*1*_) where *Z *= 0 and each (*θ*_*τ*_)_2 _ranges from 0.0 to 0.05 under the **D**_*Marquesas *_sample size configuration (Figure 6). In this case, 63% of the *Z *estimates were ≤ 1 (Figure 6A &6B). Likewise, estimates of *E*((*θ*_*τ*_)_2_) were a reliable indicator of detecting *H*_*1 *_under this special asymmetrical case of vicariance (*H*_*1*_). Even though true values of *E*((*θ*_*τ*_)_2_) ranged from 0.0 to 0.05, estimates of *E*((*θ*_*τ*_)_2_) in this case were upwardly bias, with 90% of the *E*((*θ*_*τ*_)_2_) estimates ranging from 0.21 to 0.48 (Figure 6C). Even though the *E*((*θ*_*τ*_)_2_) estimator is upwardly biased given this special case, it is upwardly biased in the direction of correct inference of *H*_*1 *_if one uses the criterion of *E*((*θ*_*τ*_)_2_) >> 0.05 to distinguish *H*_*1 *_from *H*_*2*_. In this case, our empirical estimates of *Z *and *E*((*θ*_*τ*_)_2_) given the Marquesas data were not likely the result of extreme asymmetrical soft vicariance.

The root mean square error (RMSE) from the simulation study also revealed local linear regression (LLR) to outperform the three other methods that all keep the accepted values of Z as discrete integers (LLR RMSE = 1.32; RAW RMSE = 1.90; CLR RMSE = 1.95; MPR RMSE = 2.15). However, we use all four methods for the empirical data sets to check for consistency.

## Discussion

### Model Assumptions and Robustness

While previous studies have used multi-locus population genetic data to reconstruct the demography and geography of speciation [51-55], here we use single locus mtDNA data to look at patterns across multiple co-distributed taxa. Although single locus inference can be hazardous in the face of coalescent variance and the possibility of selection, our approach offers the possibility to look at patterns of community assembly when the community consists of many non-model organisms where only "barcode" DNA sequence data can be feasibly collected. Not only does our model incorporate the stochasticity of single-locus coalescent variance across taxa, by combining datasets into a hierarchical Bayesian analysis we gain statistical "borrowing strength" [38]. The "borrowing strength" of HABC is achieved by making inferences across groups (i.e. co-distributed taxa) by pooling information across the groups without assuming the groups are from the same population [39,40]. This allows estimating congruence across groups in sub-parameters while borrowing strength from the full comparative phylogeographic sample. This "borrowing strength" translates into higher sample size depending on the magnitude of the "pooling factor" which represents the degree to which sub-parameter estimates (*Φ*) are pooled together from hyper-parameter estimates of *φ*, rather than estimated independently from each phylogeographic dataset [56].

The possibility of selection at the tightly linked mtDNA genome could bias results of our analytical method [57], especially if balancing selection occurred in the mtDNA genome in ancestral or descendent taxa such that coalescent events were much older than neutral expectations. Likewise, if positive selective sweeps occurred on the mtDNA genome after colonization or vicariance, estimates of *Z *and *E*((*θ*_*τ*_)_2_) could be biased to reflect colonization [58,59]. However, if positive selection only occurred at the mtDNA genome before colonization or vicariance, then the timing of these isolation events could be better estimated due to reduction of ancestral polymorphism. Furthermore, because selection and demography are ultimately confounding, our method will be less reliable if mitochondrial positive selection is prevalent in the comparative phylogeographic sample. This could be especially troublesome if peripatric speciation by colonization or vicariance involves positive selection at mtDNA genes that allow adaptive divergence in novel peripheral habitats [60-62]. Nonetheless, results from our HABC method can be considered conservative with respect to inferring the geographic and demographic history of isolation across a peripheral community. For example, a strong inference of colonization across an entire data set could result from both strong positive selection and/or small effective colonizing population sizes, but it would be extraordinary if strong positive selection occurred at the mtDNA genome of all *Y *co-distributed taxon-pairs.

A strong result of temporal concordance in isolation is also conservative with respect to violations from a uniform molecular clock model. If rate variation occurred across the *Y *taxa, then we would expect an inference in temporal discordance unless rates were inversely proportional with actual isolation times. Nevertheless, this HABC method will be most useful when applied to co-distributed taxon-pairs that are closely related. Our empirical application was restricted to cowrie gastropods (Cypraeidae) and the COI loci we used only marginally rejected rate constancy [63].

Although Bayesian methods are less robust if results are heavily dependent on prior bounds, results from the empirical data were not sensitive to our exploration of different prior assumptions. The overall inferences and hyper-posterior estimates from the empirical data were not sensitive to model assumptions regarding the priors of **θ **(Additional file 3) or post-isolation migration. Additionally, all four methods of post-acceptance transformation (LLR, PLR, CLR and RAW) yielded identical mode estimates of *Z *given the Marquesas data and similar mode estimates of *Z *given the Hawaii data (ranging from 4 – 5).

Another consideration is how deviations from a panmictic Wright-Fisher model could have affected our HABC estimates. Although the sampling scale is large in some of the source species (Additional file 1), the cowrie gastropod taxa we included have high dispersal capabilities and are therefore not likely to have elevated within species subdivision [63]. This is confirmed by the relatively low levels of within species average pair-wise differences (*π*_1 _and *π*_2_) in both data sets (Additional file 1). If intra-species migration rates are > 1, our idealized coalescent model assuming intra-species panmixia is somewhat appropriate (Slatkin 1985). Even with some population some structure, a standard coalescent model can suffice if a species consists of many small demes with at least moderate migration such that number of demes is approximated by a scaled effective population size [64-67]. If ancestral population structure is approximately scaled by ancestral effective population size, then our chosen priors are conservative because we allow for ancestral population sizes that are two to four times as large as the observed pair-wise distances of extant population sizes (*π*_1 _and *π*_2_; Additional file 1). However, our method should not be applied to populations that are heavily structured over large geographic scales.

### Vicariance and Dispersal in Marine Communities

Vicariance and dispersal speciation could be hugely relevant in the marine realm, especially within the highly diverse Indo-Pacific region that is dominated by islands rather than long continuous coastlines. Explanations for elevated Indo-Pacific diversity in the centrally located "coral triangle" portion of this Indo-Pacific region (Philippines, Malay Peninsula, and New Guinea) usually revolve around sympatric speciation followed by outward range shifts [68] or peripatric speciation followed by inward range shifts [69,70]. However, the plausibility of the first hypothesis of sympatric speciation is very controversial on theoretical grounds [71,72] as well as being very difficult to test empirically [18]. On the other hand, the second hypothesis of peripatric speciation is a much more likely force behind Indo-Pacific diversification if long distance oceanic dispersal to peripheral populations is sufficiently low for isolation and subsequent reproductive or ecological divergence to emerge between central and peripheral archipelagoes [1-7].

Instead of the classic vicariance model, under our marine vicariance model (or "soft vicariance") an ancestral range is inter-connected by long distance gene flow that is interrupted by oceanographic changes in temperature, sea level and/or currents [10-12]. In this case we might predict that co-distributed peripheral endemics became isolated simultaneously in taxa with lower dispersal capability. On the other hand, our marine colonization model is more similar to the classic dispersal or peripatric model. Here, allopatric isolation arises via sweepstakes colonization where the timing of colonization could be predicted to occur randomly across the co-distributed endemics after an archipelago emerges from geological processes.

### Hawaiian vs Marquesas Dynamics

Both Hawaii and the Marquesas have some of the highest levels of endemism in all of Oceania [73-75], but HABC analyses support different histories for their endemic species. The Hawaiian archipelago is the most isolated island chain in the Indo-West Pacific and has existed for > 70 My with coral reefs since at least 35 My [76]. It is now well established that terrestrial endemics can be older than the oldest emergent island in the archipelago, a pattern resulting from initial colonization of older, now subsided islands, followed by dispersal to new islands after emergence [77]. Thus, there has been ample opportunity for isolation and speciation, perhaps even more so for marine taxa. Although the lower sample sizes lead to greater uncertainty associated with the Hawaiian estimates (Figure 3), the hyper-parameter 95% credibility intervals suggest a strong inference of isolation via soft vicariance in at least a subset of the Hawaiian taxon-pairs (*Z *= 0.0 – 9.22; *E*((*θ*_*τ*_)_2_) = 0.30–0.95). If this is the case, then occupation of the Hawaiian archipelago was much older than the Marquesas archipelago, consistent with geologic evidence. Moreover, the inference of soft vicariance in a number of the taxon pairs suggests that there was greater potential for migrants between this archipelago and the central Pacific and Indo-Pacific triangle regions during older periods. Such connectivity of the Hawaiian chain to the remaining Indo-West Pacific via Johnston Atoll has been suggested in other studies [78-80].

In contrast, we find strong inference of isolation via colonization across all seven cowrie gastropod endemics co-distributed in the Marquesas, as well as a strong inference of temporal concordance in this colonization. Unlike other island chains in the Pacific Ocean that have older seamounts and atolls trending away from most recent island (e.g. Hawaii), the Marquesan hotspot is unusual in being quite young. The ages of Marquesan islands range from 1.3 My (Fatu Hiva) to 6.0 My (Eiao) [81]. If we apply a molecular clock of 1% divergence per My [63], the inferred timing of simultaneous colonization of the Marquesas archipelago is from 0.84 – 1.90 My (Additional file 3A), and is consistent with the young origins of the islands. If we accept our strong inference of temporal concordance, it could be argued that this assemblage of cowries colonized via an episodic oceanographic event that caused a surge in gene flow from the central Pacific region. Given that the Marquesas has one of the highest levels of marine endemism in Oceania [73,74], it will be interesting to see if HABC analyses on other taxon-pairs show similarly young divergences and temporal congruence.

## Conclusion

Although soft vicariance and colonization are likely to produce relatively similar genetic patterns when only a single taxon-pair is considered, our simulation analysis shows that our hierarchical Bayesian model can potentially detect if either history is a dominant process across a marine community. The empirical implementation of our method yields a strong inference of isolation via simultaneous colonization across all seven cowrie gastropod endemics co-distributed in the Marquesas. In contrast, our method shows a strong inference of isolation via soft vicariance in at least a subset of the 11 Hawaiian taxon-pairs, although the smaller sample size resulted in less certainty in our estimates.

Our HABC method exemplifies the utility in "statistical phylogeographic" approaches [82,83] rather then qualitative and descriptive approaches that make large inferences from the small details observed from gene trees [84]. The HABC approach accomplishes this by analyzing all the phylogeographic datasets at once in order to make across taxon-pair inferences about biogeographic processes while explicitly allowing for uncertainty in the demographic differences within each taxon-pair.

Although the approach described here uses HABC to test for two particular biogeographic explanations of allopatric diversification across co-distributed taxa, the HABC framework is flexible and therefore can provide a skeleton for testing other biogeographic models from comparative phylogeographic data. Indeed, one of the original objectives of phylogeography comparative was to resolve deep-seated questions about how climate change drives community assembly and evolution of whole biotas [85]. However, this goal has so far been unrealized [86-88] because comparative phylogeographic studies rarely involve more than a handful of co-distributed species. Comparative phylogeographic datasets are bound to have explosive growth as collecting DNA sequence data across a wide diversity of co-distributed taxa scales up to the level of comprehensive ecosystem sampling. Such "community-scale" comparative phylogeographic data sets could potentially test classic biogeographic hypotheses (e.g. vicariance versus dispersal) at the community level [89,90], as well as test controversial and fundamental hypotheses in community ecology such as Hubbell's Neutral theory [91], Tillman's stochastic competitive assembly model [92], and Diamond's niche assembly rules [93,94]. As comparative phylogeographic datasets grow to include > 100 co-distributed taxon-pairs, the HABC approach will be well suited to dissect temporal patterns in community assembly and thereby provide a bridge linking comparative phylogeography with community ecology.

## Abbreviations

HABC: Hierarchical Approximate Bayesian Computation; mtDNA: Mitochondrial DNA; COI: Cytochrome oxidase 1.

## Authors' contributions

MH developed, tested, and implemented the HABC model. CM collected and sequenced the cowrie data and provided feedback on model development. Both MH and CM contributed to the writing.

## Supplementary Material

Additional file 1 **Table 1 – Ranges, samples sizes and three summary statistics of cowrie data**. (A) Seven Marquesas cowrie sister taxon pairs (Cypraeidae) (B) Eleven Hawaiian cowrie sister taxon pairs. Click here for file

Additional file 2 **Table 2 – Summary of hierarchical model parameters**. Summary of the hierarchical model. Times are referred to as times before the present. (A) Sample size configuration (B) Hyper-parameters (C) Sub-parameters (D) Hyper-parameter summaries. * Estimated under the stage 1 general model. **Only estimated under a constrained stage 2 model (*Z *constrained to be the integer closest to the posterior mode estimate generated from the stage 1 analysis). Click here for file

Additional file 3 **Table 3 – Posterior mode hyper-parameter estimates and their 95% credibility intervals**. Hyper-posterior mode estimates and their 95% credibility intervals from two comparative phylogeographic data sets of taxon-pairs that include endemic species or subspecies in the (A & B) Marquesas and (C & D) Hawaiian archipelagoes. Average colonization E(*τ*_*C*_) and vicariance E(*τ*_*V*_) times are given in units of My by assuming a divergence rate of 1% per My. Bold values are obtained from stage 1 of the analysis, whereas the remaining estimates are obtained from the stage 2 analysis where *Z *and *E*((*θ*_*τ*_)_2_) are held to their estimated values obtained in stage 1. Click here for file
