# Supplementary material for: Testing comparative phylogeographic models of marine vicariance and dispersal using a hierarchical Bayesian approach
Source: BMC Evol Biol. 2008 Nov 27;8:322. doi: 10.1186/1471-2148-8-322 (PMC2614435; doi:10.1186/1471-2148-8-322)
Supplement: Additional file 1 — Table 1 – Ranges, samples sizes and three summary statistics of cowrie data. (A) Seven Marquesas cowrie sister taxon pairs (Cypraeidae) (B) Eleven Hawaiian cowrie sister taxon pairs. [file 1471-2148-8-322-S1.doc]

## Table 1 - Ranges, samples sizes and three summary statistics of cowrie data.

## (A) 7 Marquesas cowrie sister taxon pairs (Cypraeidae)

| **Marquesan Endemic**  (*n_2_*, #Samples) | **Sister Taxon**  ( *n_1_*, #Samples) | **Range of Sister** | Reciprocally  Monophyletic? | Average pairwise distances within Marquesan endemic sample | Average pairwise distances within sister taxon | Average pairwise distances between Marquesan endemic and sister taxon  *π_b_* |
| --- | --- | --- | --- | --- | --- | --- |
| *Erosaria helvola callista* (10) | *E. helvola helvola* (83) | Indo-West Pacific | N | 0.0030 | 0.007 | 0.008 |
| *Purpuradusta fimbriata marquesana* (8) | *P. fimbriata unifasciata* (51) | Pan-Pacific | N | 0.0189 | 0.018 | 0.020 |
| *Mauritia maculifera martybealsi* (10) | *M. maculifera scindata* (19) | Southeast Polynesia | Y | 0.0013 | 0.002 | 0.031 |
| *Lyncina carneola propinqua* (6) | *L. carneola carneola* (15) | Indo-West Pacific | Y | 0.0038 | 0.004 | 0.020 |
| *Cypraea tigris* (5) | *C. tigris* (55) | Pan-Pacific | N | 0.0088 | 0.010 | 0.019 |
| *Cribrarula astaryi* (10) | *C. cumingii* (12) | Societies/Tuamotu | Y | 0.0068 | 0.006 | 0.028 |
| *Erosaria thomasi* (5) | *E. kingae* (1) | Societies/Pitcairn | Y | 0.0072 | N/A | 0.015 |

## (B) 11 Hawaiian cowrie sister taxon pairs

| **Hawaiian Endemic**  (*n_2_*, #Samples) | **Sister Taxon**  ( *n_1_*, #Samples) | **Range of Sister** | Reciprocally  Monophyletic? | Average pairwise distances within Hawaiian endemic sample | Average pairwise distances within sister taxon | Average pairwise distances between Marquesan endemic and sister taxon  *π_b_* |
| --- | --- | --- | --- | --- | --- | --- |
| *Nucleolaria granulata (3)* | *N. nucleus (9)* | Indo-West Pacific | N | 0.013 | 0.010 | 0.011 |
| *Erosaria helvola hawaiiensis (12)* | *E. helvola helvola (81)* | Indo-West Pacific | N | 0.005 | 0.006 | 0.011 |
| *Monetaria caputserpentis caputophidii (10)* | *M. caputserpentis caputserpentis (14)* | Indo-West Pacific | Y | 0.008 | 0.007 | 0.008 |
| *Purpuradusta fimbriata waikikiensis (7)* | *Purpuradusta fimbriata unifasciata (44)* | Pan-Pacific | N | 0.006 | 0.019 | 0.019 |
| *Pustularia mauiensis (6)* | *Pustularia bistrinotata (5)* | Indo-West Pacific | Y | 0.022 | 0.006 | 0.053 |
| *Staphylaea semiplota (1)* | *Staphylaea staphylaea staphylaea (10)* | Pan-Pacific | Y | N/A | 0.001 | 0.065 |
| *Talostolida rashleighana rashleighana (1)* | *Talostolida rashleighana pseudoteres (1)* | West Pacific | Y | N/A | N/A | 0.047 |
| *Talostolida latior (1)* | *Talostolida subteres (6)* | Tuamotu/Societies | Y | N/A | 0.001 | 0.081 |
| *Cribrarula gaskoini (11)* | *Cribrarula catholicorum (3)* | West Pacific | Y | 0.005 | 0.008 | 0.074 |
| *Luria tessellata (5)* | *Luria isabella (25)* | Indo-West Pacific | Y | 0.008 | 0.010 | 0.115 |
| *Lyncina sulcidentata (2)* | *Lyncina schilderorum (6)* | Pan-Pacific | Y | 0.005 | 0.008 | 0.011 |
