# Supplementary material for: Testing comparative phylogeographic models of marine vicariance and dispersal using a hierarchical Bayesian approach
Source: BMC Evol Biol. 2008 Nov 27;8:322. doi: 10.1186/1471-2148-8-322 (PMC2614435; doi:10.1186/1471-2148-8-322)
Supplement: Additional file 2 — Table 2 – Summary of hierarchical model parameters. Summary of the hierarchical model. Times are referred to as times before the present. (A) Sample size configuration (B) Hyper-parameters (C) Sub-parameters (D) Hyper-parameter summaries. * Estimated under the stage 1 general model. **Only estimated under a constrained stage 2 model (Z constrained to be the integer closest to the posterior mode estimate generated from the stage 1 analysis). [file 1471-2148-8-322-S2.doc]

## Table 2 - Summary of hierarchical model parameters.

Summary of the hierarchical model. Times are referred to as times before the present. (A) Sample size configuration (B) Hyper-parameters (C) Sub-parameters (D) Hyper-parameter summaries. * Estimated under the stage 1 general model. **Only estimated under a constrained stage 2 model (*Z* constrained to integer closest to the posterior mode estimate generated from the stage 1 analysis).

(A)

**Sample Size** Description

**Variables**

*Y* Number of taxon-pairs

$n_{1}$ = {$n_{1}^{1}$, …,$n_{1}^{Y}$} Vector of sample sizes of central taxon (number of individuals)

$n_{2}$ = {$n_{2}^{1}$, …,$n_{2}^{Y}$} Vector of sample sizes of peripheral taxa (number of individuals)

(B)

**Hyper- Parameters Description Hyper-Prior**

| *Z* * | The number of descendent populations per *Y* taxon-pairs  that arise by colonization at times *T_C_* = {$t_{C}^{1}$*,…,*$t_{C}^{Z}$} | **Stage 1:** [0, *Y*] discrete uniform  **Stage 2:** Fixed |
| --- | --- | --- |
| Ψ*_C_* ** | The number of *different* colonization times $Y_{C}$ = {$t_{C}^{1}$, …, $t_{C}^{Y_{C}}$} across *Z* *actual* colonization times *T_C_* = {$t_{C}^{1}$*,…,*$t_{C}^{Z}$}. | [1,*Z*] discrete uniform | Z > 0  Ψ*_C_* = N/A | Z = 0 |
| Ψ*_V_* ** | The number of *different* vicariance times $Y_{V}$ = {$t_{V}^{1}$, … $t_{V}^{Y_{V}}$} across (*Y–Z)* *actual* vicariance times *T_V_* = {$t_{V}^{1}$*,…,*$t_{V}^{(Y-Z)}$} | [1,(*Y*-*Z*)\ discrete uniform | Z < *Y*  Ψ*_V_* = N/A | Z = *Y* |

(C)

Sub-parameters (general) Description Prior

| $q$ = {$q^{1}$, …,$q^{Y}$} | Summed population mutation-drift parameter, where each taxon-pair’s $q^{i}$ is 2*N^i^μ^i^* . *N^i^* is the summed haploid effective population size of the *i*th taxon-pair with daughter populations $N_{1}^{i}$ *+* $N_{2}^{i}$, and *μ* is the per gene per generation mutation rate | Uniform [0.0, *θ_MAX_*] |
| --- | --- | --- |
| $q_{1}$ = {$q_{1}^{1}$, …,$q_{1}^{Y}$} | Population mutation-drift parameter for source daughter populations (colonization model) or central daughter populations (vicariance model) at *τ^i^* = 0 | Uniform [0.0, 2$q^{i}$] |
| $q_{2}$ = {$q_{2}^{1}$, …,$q_{2}^{Y}$}, | Population mutation-drift parameter for peripheral daughter populations at *τ ^i^* = 0 | Uniform [0.0, (2$q^{i}$ - $q_{1}^{i}$)] |

(D)

**Sub-parameters (soft vicariance; *H_1_***) Description Prior

| $(q_{t})_{1}$ = {$(q_{t})_{1}^{1}$, …, $(q_{t})_{1}^{(Y-Z)}$} | Relative effective population size for central population until $t_{V}^{i}$. At $t_{V}^{i}$, $(q_{t})_{1}^{i}$ exponentially grows until reaching size $q_{1}^{i}$ at $t_{V}^{i}$ = 0. Units are in relative size of $q^{i}$ | Uniform [0.5, 1.0] |
| --- | --- | --- |
| $(q_{t})_{2}$ = {$(q_{t})_{2}^{1}$, …, $(q_{t})_{2}^{(Y-Z)}$} | Relative effective population size for peripheral population until $t_{V}^{i}$. At $t_{V}^{i}$,$(q_{t})_{2}^{i}$ exponentially grows until reaching size $q_{2}^{i}$ at $t_{V}^{i}$= 0. Units are in relative size of $q^{i}$ | Uniform [0.0, 1.0] |
| $Y_{V}$ = {$t_{V}^{1}$, … $t_{V}^{Y_{V}}$} | Matrix of Ψ*_V_ different* vicariance times. Each $t_{V}^{i}$ is in units of $q_{\text{AVE}}$/μ generations where $q_{\text{AVE}}$is the parametric expectation of *θ* across *Y* taxon-pairs given the prior distribution and μ is the per gene per generation mutation rate | Each *t* drawn from uniform [0.0, 5.0] |
| *T_V_* = {$t_{V}^{1}$*,…,*$t_{V}^{(Y-Z)}$} | Matrix of *actual* vicariance times. Each $t_{V}^{i}$is in units of $q^{i}$/μ generations, where $q^{i}$ is each taxon-pair’s population mutation-drift parameter and μ is the per gene per generation mutation rate | Vicariance times $t_{V}^{1}$, …, $t_{V}^{Y_{V}}$ sequentially assigned to $t_{V}^{1}$, … $t_{V}^{Y_{V}}$. Remaining elements of *T_V_* (($t_{V}^{(Y_{V}+1)}$*,…,*$t_{V}^{(Y-Z)}$)) are assigned by randomly drawing with replacement from $Y_{V}$ matrix of *different* times ({$t_{V}^{1}$, …, $t_{V}^{Y_{V}}$). |
| $M_{1}$ = {$M_{1}^{1}$, …, $M_{1}^{(Y-Z)}$} | Number of effective migrants per generation between central and peripheral populations ($n_{1}^{i}$ and $n_{2}^{i}$) until $t_{V}^{i}$ | Uniform [0.0, 100.0] |
| $M_{2}$ = {$M_{2}^{1}$, …, $M_{2}^{(Y-Z)}$} | Number of effective migrants per generation between central and peripheral populations at $t_{V}^{i}$ and until τ = 0. | Uniform [0.0, 1.0] |

**Sub-parameters (colonization; *H_2_*) Description Prior**

| $(q_{t})_{1}$ = {$(q_{t})_{1}^{1}$, …, $(q_{t})_{1}^{Z}$} | Relative effective population size for source daughter population until colonization time $t_{C}^{i}$. At $t_{C}^{i}$, $(q_{t})_{1}^{i}$ exponentially grows until reaching size $q_{1}^{i}$ at $t_{C}^{i}$= 0. Units are in relative size of $q^{i}$ | Uniform [0.5, 1.0] |
| --- | --- | --- |
| $(q_{t})_{2}$ = {$(q_{t})_{2}^{1}$, …, $(q_{t})_{2}^{Z}$} | Relative effective population size for colonized peripheral daughter population at time of colonization $t_{C}^{i}$. At $t_{C}^{i}$, $(q_{t})_{2}^{i}$ exponentially grows until reaching size $q_{2}^{i}$ at $t_{C}^{i}$ = 0. Units are in relative size of $q^{i}$ | Uniform [0.0, 0.05] |
| $Y_{C}$ = {$t_{C}^{1}$, …, $t_{C}^{Y_{C}}$} | Matrix of Ψ*_C_ different* colonization times. Each $t_{C}^{i}$ is in units of $q_{\text{AVE}}$/μ generations and $q_{\text{AVE}}$is the parametric expectation of *θ* across *Y* taxon-pairs given the prior distribution | Each *t* drawn from uniform [0.0, 5.0] |
| *T_C_* = {$t_{C}^{1}$*,…,*$t_{C}^{Z}$ } | Matrix of *Z* *actual* colonization times. Each $t_{C}^{i}$is in units of $q^{i}$/μ generations, where $q^{i}$ is each taxon-pair’s population mutation-drift parameter and μ is the per gene per generation mutation rate | Colonization times $t_{C}^{1}$, …, $t_{C}^{Y_{C}}$ sequentially assigned to $t_{C}^{1}$, … $t_{C}^{Y_{C}}$. Remaining elements of *T_C_* (($t_{C}^{(Y_{C}+1)}$*,…,*$t_{C}^{Z}$)) are assigned by randomly drawing with replacement from $Y_{C}$ matrix of *different* times ({$t_{C}^{1}$, …, $t_{C}^{Y_{C}}$). |

(E) Descriptions of sub-parameter summaries. *Estimated under the general model. **Only estimated under a constrained model (*Z* constrained to be its maximum posterior estimate generated under the general model).

Sub-parameter summaries Description Prior Bounds

| *E*($(q_{t})_{2}$) * | The average relative effective population size of *Z* colonized populations and (*Y-Z*) peripheral populations that become isolated at *τ_C_* and/or *τ_V_* | [0.0, 1.0] |
| --- | --- | --- |
| E(*τ_C_*) ** | The mean colonization time *τ_C_* across *Z* colonized populations | [0.0, 5.0] |
| E(*τ_V_*) ** | The mean vicariance time *τ_V_*across (*Y-Z*) taxon-pairs that diverge by vicariance (gene flow disruption) | [0.0, 5.0] |
| *C* ** | Var(*τ_C_*)/ E(*τ_C_*) | [0.0, 5.0] |
| *V* ** | Var(*τ_V_*)/ E(*τ_V_*) | [0.0, 5.0] |
