# Supplementary material for: Testing comparative phylogeographic models of marine vicariance and dispersal using a hierarchical Bayesian approach
Source: BMC Evol Biol. 2008 Nov 27;8:322. doi: 10.1186/1471-2148-8-322 (PMC2614435; doi:10.1186/1471-2148-8-322)
Supplement: Additional file 3 — Table 3 – Posterior mode hyper-parameter estimates and their 95% credibility intervals. Hyper-posterior mode estimates and their 95% credibility intervals from two comparative phylogeographic data sets of taxon-pairs that include endemic species or subspecies in the (A & B) Marquesas and (C & D) Hawaiian archipelagoes. Average colonization E(τC) and vicariance E(τV) times are given in units of My by assuming a divergence rate of 1% per My. Bold values are obtained from stage 1 of the analysis, whereas the remaining estimates are obtained from the stage 2 analysis where Z and E((θτ)2) are held to their estimated values obtained in stage 1. [file 1471-2148-8-322-S3.doc]

## Table 3 - Posterior mode hyper-parameter estimates and their 95% credibility intervals

Hyper-posterior mode estimates and their 95% credibility intervals from two comparative phylogeographic data sets of taxon-pairs that include endemic species or subspecies in the (A & B) Marquesas and (C & D) Hawaiian archipelagoes. Average colonization E(*τ_C_*) and vicariance E(*τ_V_*) times are given in units of My by assuming a divergence rate of 1% per My. **Bold** values are obtained from stage one of the analysis, whereas the remaining estimates are obtained from the stage two analysis where *Z* and *E*($(q_{t})_{2}$) are held to their estimated values obtained in stage one.

(A) Marquesas; prior upper bound of *θ* (*θ_MAX_*) = 25.0

Hyper- Tolerance = 0.0005 Tolerance = 0.001

Parameters quantiles quantiles

|  | Estimate | 0.025 | 0.975 | Estimate | 0.025 | 0.975 |
| --- | --- | --- | --- | --- | --- | --- |
| *Z* | **7.00** | **3.42** | **7.00** | **7.00** | **3.23** | **7.00** |
| *E*($(q_{t})_{2}$) | **0.00** | **0.00** | **0.37** | **0.07** | **0.00** | **0.31** |
| Ψ*_C_* | 1.00 | 1.00 | 2.46 | 1.10 | 1.00 | 2.34 |
| *C* | 0.00 | 0.00 | 0.17 | 0.00 | 0.00 | 0.13 |
| E(*τ_C_*) | 1.58  My | 0.86  My | 1.90  My | 1.36  My | 0.84  My | 1.76  My |

(B) Marquesas; prior upper bound of *θ* (*θ_MAX_*) = 50.0

Hyper- Tolerance = 0.0005 Tolerance = 0.001

Parameters quantiles quantiles

|  | Estimate | 0.025 | 0.975 | Estimate | 0.025 | 0.975 |
| --- | --- | --- | --- | --- | --- | --- |
| *Z* | **6.92** | **1.26** | **7.00** | **6.99** | **1.85** | **7.00** |
| *E*($(q_{t})_{2}$) | **0.00** | **0.00** | **0.38** | **0.00** | **0.00** | **0.36** |
| Ψ*_C_* | 1.00 | 1.00 | 2.46 | 1.03 | 1.00 | 3.46 |
| *C* | 0.00 | 0.00 | 0.17 | 0.00 | 0.00 | 0.22 |
| E(*τ_C_*) | 1.58  My | 0.86  My | 1.90  My | 1.38  My | 0.82  My | 1.92  My |

(C) Hawaii; prior upper bound of *θ* (*θ_MAX_*) = 50.0

Hyper- Tolerance = 0.0005 Tolerance = 0.001

Parameters quantiles quantiles

|  | Estimate | 0.025 | 0.975 | Estimate | 0.025 | 0.975 |
| --- | --- | --- | --- | --- | --- | --- |
| *Z* | **3.96** | **0.00** | **9.22** | **3.96** | **0.01** | **9.14** |
| *E*($(q_{t})_{2}$) | **0.71** | **0.31** | **0.95** | **0.72** | **0.30** | **0.93** |
| Ψ*_C_* | 1.77 | 1.26 | 4.00 | 1.72 | 1.08 | 4.15 |
| *C* | 0.21 | 0.05 | 1.11 | 0.11 | 0.00 | 0.86 |
| E(*τ_C_*) | 2.74  My | 1.00  My | 4.76 My | 2.46  My | 1.06  My | 4.62  My |
| Ψ*_V_* | 1.15 | 1.00 | 7.00 | 1.19 | 1.00 | 8.11 |
| *V* | 0.20 | 0.00 | 2.23 | 0.20 | 0.01 | 2.00 |
| E(*τ_V_*) | 3.08  My | 0.00  My | 7.16  My | 3.00  My | 0.16  My | 7.20  My |

(D) Hawaii; prior upper bound of *θ* (*θ_MAX_*) = 100.0

Hyper- Tolerance = 0.0005 Tolerance = 0.001

Parameters quantiles quantiles

|  | Estimate | 0.025 | 0.975 | Estimate | 0.025 | 0.975 |
| --- | --- | --- | --- | --- | --- | --- |
| *Z* | **3.82** | **0.00** | **9.43** | **3.98** | **0.00** | **9.61** |
| *E*($(q_{t})_{2}$) | **0.76** | **0.28** | **0.96** | **0.57** | **0.23** | **0.95** |
| Ψ*_C_* | 1.73 | 1.16 | 5.29 | 1.83 | 1.08 | 4.42 |
| *C* | 0.26 | 0.04 | 1.01 | 0.16 | 0.01 | 0.91 |
| E(*τ_C_*) | 2.74  My | 1.00  My | 4.76 My | 2.46  My | 1.06  My | 4.62  My |
| Ψ*_V_* | 1.41 | 1.02 | 7.00 | 1.09 | 1.06 | 8.19 |
| *V* | 0.21 | 0.01 | 2.26 | 0.26 | 0.00 | 1.05 |
| E(*τ_V_*) | 3.05  My | 0.00  My | 7.32  My | 2.99  My | 0.12  My | 7.37  My |
